# Menaquinone-4 enhances testosterone production in rats and testis-derived tumor cells

**DOI:** 10.1186/1476-511X-10-158

**Published:** 2011-09-13

**Authors:** Asagi Ito, Hitoshi Shirakawa, Naofumi Takumi, Yoshihiko Minegishi, Ai Ohashi, Zakir H Howlader, Yusuke Ohsaki, Toshiro Sato, Tomoko Goto, Michio Komai

**Affiliations:** 1 Laboratory of Nutrition, Graduate School of Agricultural Science, Tohoku University, Sendai 981-8555, Japan; 2 Department of Biochemistry, University of Dhaka, Dhaka, Bangladesh; 3 Fine Chemical & Food Laboratories, J-Oil Mills, Inc., Fukuroi 437-1111, Japan

**Keywords:** I-10 cells, menaquinone-4, protein kinase A, testis, testosterone, vitamin K

## Abstract

**Background:**

Vitamin K is essential for the posttranslational modification of various Gla proteins. Although it is widespread in several organs, including the testis, the function of vitamin K in these organs is not well characterized. In this study, we investigated the function of vitamin K in the testis and analyzed its role in steroidogenesis.

**Methods:**

Eight-week-old male Wistar rats were fed a diet supplemented with menaquinone-4 (MK-4, 75 mg/kg diet), one of the predominant K_2 _vitamins present in the testis, for 5 weeks. *In vivo *testosterone levels of the rats' plasma and testes were measured by enzyme-linked immunosorbent assay, and *in vitro *testosterone levels of testis-derived tumor cells (I-10 cells) maintained in Ham's F-10 medium with 10% fetal bovine serum were measured following treatment with MK-4 (0 to 100 μM) at several time points. Testosterone and cellular protein levels were analyzed with respect to their effects on steroidogenesis.

**Results:**

Testosterone levels in the plasma and testes of MK-4-fed rats were significantly increased compared to those of control rats, with no obvious differences in plasma luteinizing hormone levels. Secreted testosterone levels from I-10 cells were elevated by MK-4, but not by vitamin K_1_, in a dose-dependent manner independent of cAMP treatment. Western blot analysis revealed that expression of CYP11A, the rate-limiting enzyme in steroidogenesis, and phosphorylation levels of protein kinase A (PKA) and the cAMP response element-binding protein were all stimulated by the presence of MK-4. Enhancement of testosterone production was inhibited by H89, a specific inhibitor of PKA, but not by warfarin, an inhibitor of γ-glutamylcarboxylation.

**Conclusions:**

MK-4 stimulates testosterone production in rats and testis-derived tumor cells via activation of PKA. MK-4 may be involved in steroidogenesis in the testis, and its supplementation could reverse the downregulation of testosterone production in elders.

## Background

Vitamin K acts as the cofactor of γ-glutamylcarboxylase, which converts specific glutamate residues into γ-carboxyglutamate (Gla) in blood coagulation factors and bone matrix proteins [1,2]. Two types of naturally-occurring vitamin K molecules have been identified: phylloquinone (vitamin K_1_) and menaquinones (vitamin K_2_). Vitamin K_1 _is synthesized and stored in green vegetables, whereas vitamin K_2 _is mainly produced by microorganisms and is enriched for in fermented foods. Menaquinone-4 (MK-4), an analogue of vitamin K_2_, contains a geranylgeranyl group (four isoprene units) as a side chain and is obtained from the conversion of vitamin K_1 _and other menaquinones in various animal tissues and cultured cells [3-8]. In rodents, MK-4 is observed in not only the liver and bone, but in organs such as the brain, pancreas, and gonadal tissues as well. Novel functions of vitamin K_1 _and MK-4 have been reported recently [1], but the detailed mechanism and physiological significance of the conversion of vitamin K_1 _to MK-4 in various tissues remains to be elucidated.

The testis consists of three main cell types, each with specific functions: i) spermatogonia and its differentiated cells, which are located in the seminiferous tubules; ii) Leydig cells, which produce sex hormones and are distributed in the connective tissue of the convoluted seminiferous tubules; and iii) Sertoli cells, which form the basement membrane of the seminiferous tubules and offer the environment necessary for the differentiation and maturation of germ cells [9]. Leydig cells synthesize and secrete testosterone and are dependent on luteinizing hormone (LH), which is secreted from the pituitary gland. The LH receptor, a G-protein-coupled receptor located on the surface membrane of Leydig cells, stimulates adenylate cyclase and elevates intracellular cyclic-AMP (cAMP) levels after interaction with LH, followed by activation of protein kinase A (PKA) and other steroidogenic proteins. Steroidogenic acute regulatory protein (StAR) transports cholesterol into the inner membrane of mitochondria, and the enzyme CYP11A catalyzes the production of pregnenolone from transported cholesterol; these two proteins control key steps in the conversion of cholesterol to testosterone [10].

In a previous study, we performed a comprehensive gene expression analysis to elucidate the functions of vitamin K in the testis [11] and found that mRNA levels of steroidogenic genes were significantly reduced in the testis of vitamin K-deficient rats, accompanied by low testosterone levels in the rats' testis and plasma. In this current study, we further explored the effects of vitamin K on testosterone production in rat testes and tumor-derived Leydig cells.

## Methods

### Materials

MK-4 and vitamin K_1 _were obtained from Nisshin Pharma Inc. (Tokyo, Japan) and Wako Pure Chemicals (Osaka, Japan), respectively. All vitamin K analogues were dissolved in ethanol at 20 mM and stored at -20°C with protection from light until used to maintain their stability. When vitamin K was added to the cell culture medium, the final concentration of ethanol as solvent was adjusted to 0.5%. Dibutyryl cyclic-AMP (db-cAMP), forskolin (FSK), and *N*-[2-(*p*-bromocinnamylamino) ethyl]-5-isoquinoline sulfonamide (H89), which is a protein kinase A (PKA)-specific inhibitor, were purchased from Sigma (St. Louis, MO), and warfarin potassium was obtained from Eisai Co. Other reagents were purchased from Wako Pure Chemicals.

### Animals and diets

Male Wistar rats were purchased from Japan SLC, (Shizuoka, Japan) and kept in a specific pathogen-free unsterilized animal house. Rats were maintained on an open formula, non-purified diet (Funabashi Farms Co., Japan) until the start of the experiment, at which time they were 8 weeks of age. All animals were housed in individual cages, each with a wire-mesh floor, at 23°C ± 2°C on a 12:12 light:dark cycle (lights on at 8:00). Control (Cont) and MK-4-supplemented (MK-4 sup) diets were prepared by adding vitamin K_1 _or MK-4 to the vitamin K-free AIN-93G-based standard diet (final concentrations of 0.75 and 75 mg/kg diet, respectively). The concentration of vitamin K_1 _in the Cont diet was based on the AIN-93G formula [12].

### Ethical guidelines

The experimental plan for the present study was approved by the Animal Research Animal Care Committee of the Graduate School of Agricultural Science, Tohoku University. All experiments followed the guidelines issued by that committee in accordance with the Japanese government legislation (1980), which supervised the care and use of the rats in the present study.

### Cell culture

Leydig tumor cells from mice (I-10) and rats (R2C) were obtained from the Health Science Research Resources Bank (Osaka, Japan) and maintained in Ham's F-10 medium (Sigma) supplemented with 10% fetal bovine serum (Biowest, Nuaillé, France), 50 U/mL penicillin, and 50 mg/mL streptomycin at 37°C in a 5% CO_2 _atmosphere. Both cell types were seeded into12-well plates at a density of 6.0 × 10^4 ^cells per well and incubated overnight. The culture medium was then replaced with fresh medium, and MK-4 was added to the cells at final concentrations of 0, 3, 10, 30, or 100 μM.

### Measurement of testosterone and luteinizing hormone

Cell culture media of I-10 and R2C cells were collected and centrifuged at 1,000 × *g *for 5 min. Concentrations of testosterone in the supernatants were determined using the testosterone EIA kit (Cayman Chemical, Ann Arbor, MI). Testosterone in the rats' plasma and testis was extracted twice in five volumes of diethyl ether. After extraction, the organic phase was collected by centrifugation at 1,500 × *g *for 5 min and evaporated using a vacuum centrifugal evaporator. The extract was resuspended in an adequate volume of EIA buffer from the kit. The luteinizing hormone (LH) concentration in the plasma was determined using a rodent LH enzyme-linked immunosorbent assay (ELISA) test kit purchased from Endocrine Technologies (Newark, CA).

### Cell growth assays

I-10 cells were seeded into 96-well plates at a density of 1.2 × 10^4 ^cells/well. The medium was changed the following day, and MK-4 was added to final concentrations of 0, 3, 10, 30, or 100 μM. Following incubation for 24 h, the number of viable cells in each sample was determined using the Premix WST-1 Cell Proliferation Assay System (Takara Bio Inc., Shiga, Japan) according to the manufacturer's instructions.

### Western blot analysis

Whole cell extracts of I-10 cells were prepared using lysis buffer (50 mM HEPES-NaOH (pH 7.5), 150 mM NaCl, 10% glycerol, 1% Triton X-100, and 1.5 mM MgCl_2_) containing proteinase inhibitors (Complete Proteinase Inhibitor Cocktail, Roche Applied Science, Mannheim, Germany) and phosphatase inhibitors (PhosSTOP Phosphatase Inhibitor Cocktail, Roche Applied Science). Rat testis tissue was homogenized in PBS buffer containing 1 mM phenylmethylsulfonyl fluoride. The protein concentration in the lysate was measured with a protein assay reagent (Bio-Rad, Hercules, CA). The proteins were denatured in an SDS gel-loading buffer, resolved by 10% SDS-polyacrylamide gel electrophoresis, and transferred onto a polyvinylidene fluoride membrane (Millipore, Billerica, MA). After blocking for 1 h with TBS-T (10 mM Tris-HCl pH 7.4, 150 mM NaCl, and 0.1% Tween 20) containing 5% bovine serum albumin (Sigma), the membrane was incubated with anti-phospho PKA, anti-PKA, anti-phospho CREB (Cell Signaling Technology, Danvers, MA), anti-CREB (Sigma), anti-StAR (Affinity Bioreagents, Golden, CO), and anti-CYP11A (Chemicon, Temecula, CA) and then visualized with the Immobilon Western reagent (Millipore) using a LAS-4000 mini luminescent image analyzer (Fujifilm, Tokyo, Japan). The relative expression level of each protein was normalized according to the amount of α-tubulin or β-actin detected by their respective antibodies (Sigma and Abcam, Tokyo, Japan).

### Measurement of vitamin K content in cultured cells and organs by HPLC

Cultured cells and tissue samples were homogenized in five volumes of 66% 2-propanol. Vitamin K was then extracted from the homogenate with six volumes of n-hexane as previously described [13] and measured using a fluorescence-HPLC system (Waters 600E system; Puresil 5C18 column, Waters, Milford, MA; RC 10-3 PtO_2 _column, Shiseido-Irica, Kyoto, Japan; Hitachi F-1000 fluorescence detector, excitation at 240 nm, emission at 430 nm; Hitachi D-2000 data processor, Tokyo, Japan). The vitamin K_1 _and MK-4 concentrations in the tissues were determined by measuring their relative fluorescence intensities using menaquinone-3 (MK-3) as an internal standard [11,13]. Vitamin K levels in cultured cells were determined by the same procedure except that menaquinone-5 (MK-5) was used as an internal standard instead of MK-3. MK-3 and MK-5 were obtained from Eisai Co. (Tokyo, Japan)

### Statistical analysis

**Figure 1:**
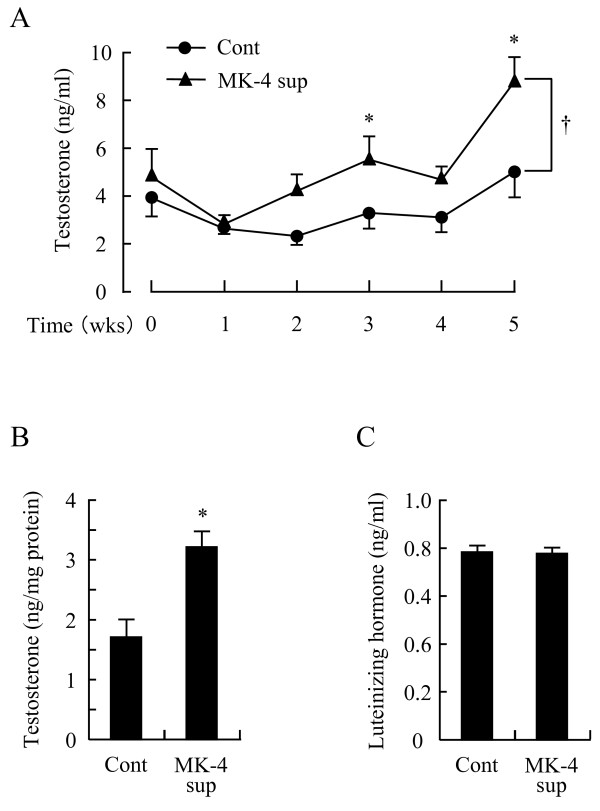
**Menaquinone-4 supplemented diet enhances testosterone production in rat testis**. Male Wistar rats were fed with a control (Cont) or a MK-4-supplemented (MK-4 sup) diet for 5 weeks. Testosterone concentrations in the plasma from the tail vein (A) and testis (B) and luteinizing hormone levels in plasma from the abdominal aorta (C) were measured by ELISA. Data are represented as means ± SE (n = 7 or 8). Values are significantly different from those of the control group at *p < 0.05. †Plasma testosterone levels in the MK-4 sup group were significantly increased when compared with the control group (*p *< 0.05).

**Figure 2:**
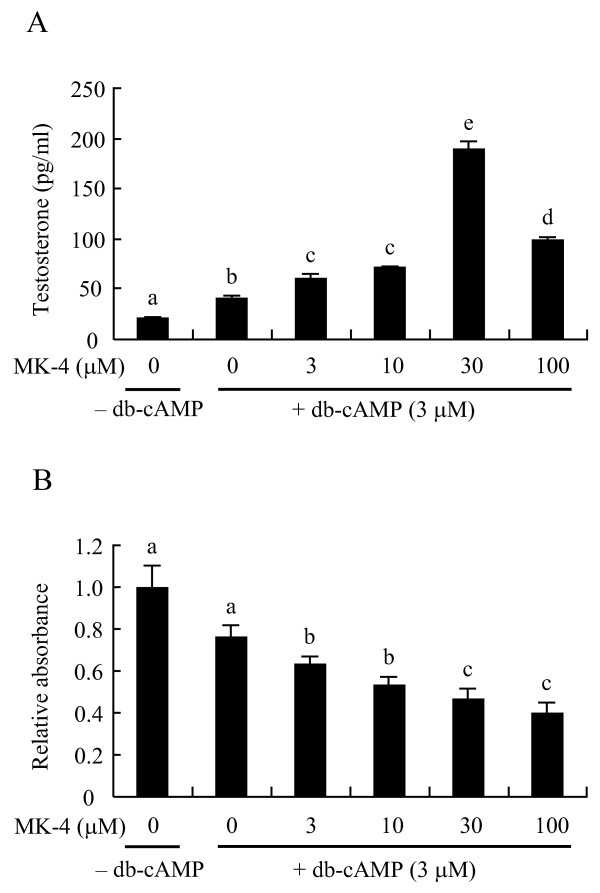
**Menaquinone-4 enhances testosterone production in I-10 cells in the presence of cAMP**. Mouse testis tumor-derived I-10 cells were treated with dibutyryl-cAMP (db-cAMP, 3 μM) and various concentrations of menaquinone-4 (MK-4) for 24 h. (A) Testosterone levels in cultured medium were determined by ELISA. Data are represented as means ± SE (n = 3). (B) The cell proliferation assay was performed using the WST-1 assay kit. Data are represented as means of relative absorbance values at 450 nm ± SE (n = 3). Values with different letters are significantly different at *p *< 0.05.

**Figure 3:**
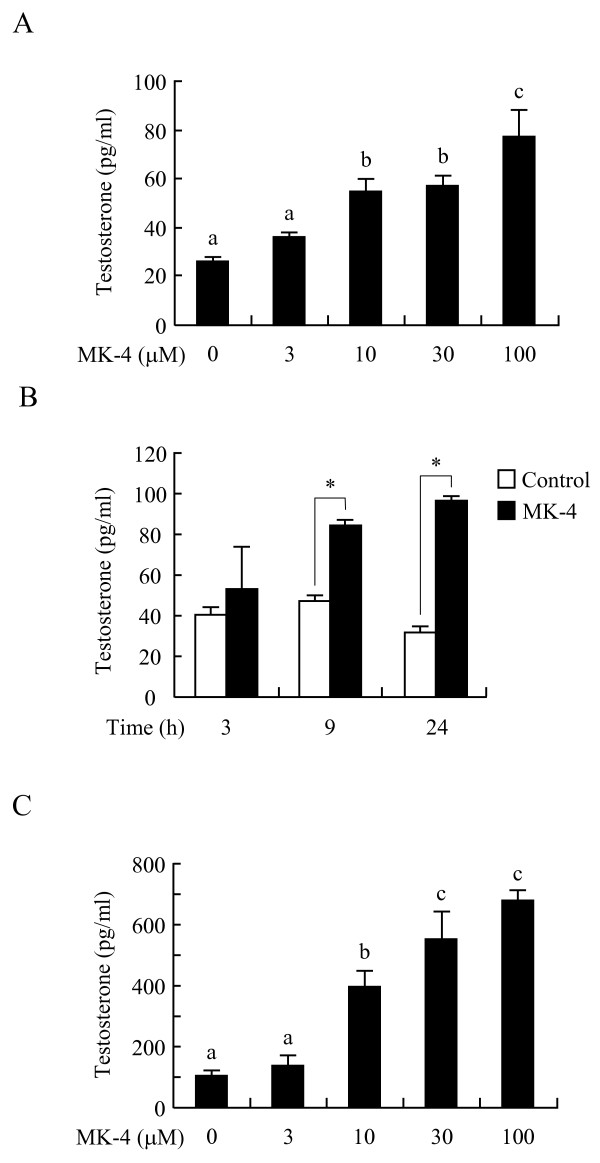
**Menaquinone-4 enhances testosterone production in I-10 and R2C cells without cAMP stimulation**. I-10 cells were treated with various concentrations of menaquinone-4 (MK-4) for 24 h (A) or 30 μM MK-4 for different time periods (B). Rat testis tumor-derived R2C cells were treated with various concentrations of MK-4 for 24 h (C). Testosterone levels in the culture medium were determined by ELISA. Data are represented as means ± SE (n = 3). Values indicated with different letters in A and C are significantly different at *p *< 0.05. Values indicated by an asterisk in B are significantly different at *p *< 0.05.

**Figure 4:**
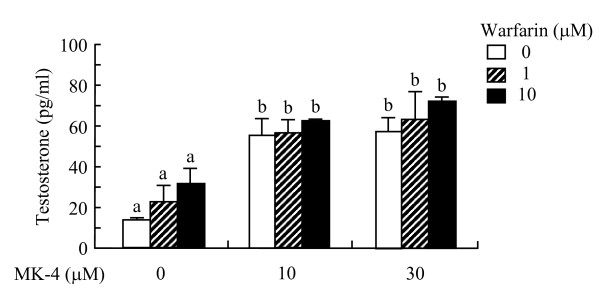
**Warfarin does not inhibit enhanced testosterone production by menaquinone-4 in I-10 cells**. I-10 cells were treated with warfarin and menaquinone-4 (MK-4) simultaneously for 24 h, and testosterone levels in the culture medium were then determined by ELISA. Data are represented as means ± SE (n = 3). Values indicated with different letters are significantly different at *p *< 0.05.

**Figure 5:**
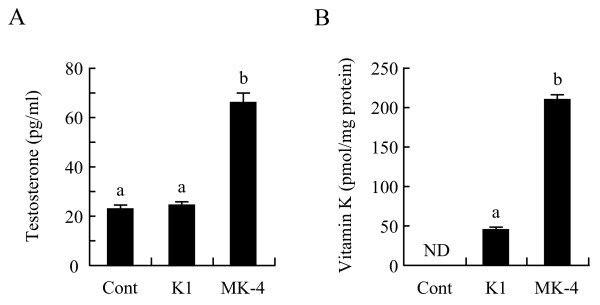
**Menaquinone-4, but not vitamin K_1_, enhances testosterone production in I-10 cells**. I-10 cells were treated with 10 μM vitamin K_1 _(K1), or menaquinone-4 (MK-4) for 24 h, and testosterone levels in the culture medium were determined by ELISA (A), or I-10 cells were treated with K1 or MK-4 for 3 h, and vitamin K levels in cells were determined by fluorescent-HPLC (B). Data are represented as means ± SE (n = 3). Values with different letters are significantly different at *p *< 0.05. ND, not detected.

**Figure 6:**
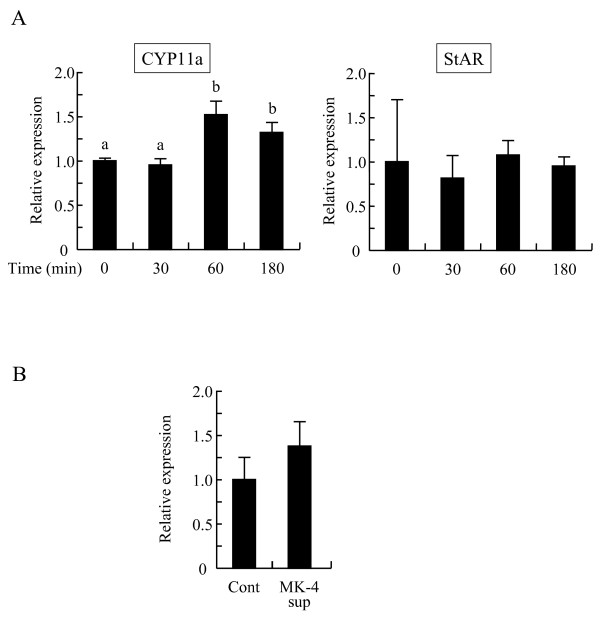
**Menaquinone-4 increases CYP11A protein levels in I-10 cells**. I-10 cells were treated with menaquinone-4 (MK-4) for the indicated time. After the treatment, whole cell extract was prepared, and CYP11A and StAR levels were measured by Western blot analysis (A). Whole cell extracts from the testis of rats fed control (Cont) or MK-4 supplemented (MK-4 sup) diets for 5 weeks were prepared, and CYP11A levels were measured by Western blot analysis (B). Data are represented as mean ± SE (n = 3) normalized to α-tubulin (A) or β-actin levels (B) and expressed as a fold-increase/decrease of values compared to the MK-4 control cells or the control diet group. Values with different letters are significantly different at *p *< 0.05.

**Figure 7:**
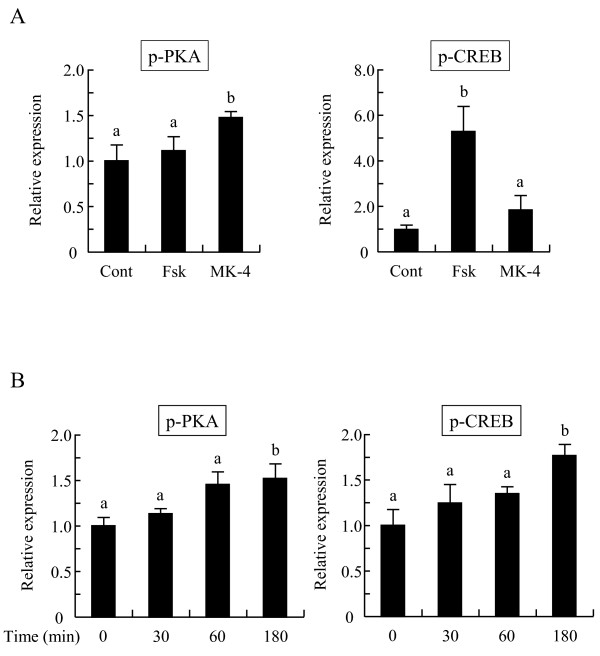
**Menaquinone-4 stimulates the protein kinase A pathway in I-10 cells**. I-10 cells were treated with menaquinone-4 (MK-4) or forskolin (Fsk) for 2 h. After the treatment, whole cell extracts were prepared, and the phosphorylation levels of protein kinase A (p-PKA) and CREB (p-CREB) were measured by Western blot analysis (A). I-10 cells were treated with MK-4 for the indicated time. After the treatment, p-PKA and p-CREB levels in the whole cell extract were measured by Western blot analysis (B). Data are represented as mean ± SE (n = 3), normalized to α-tubulin levels and expressed as a fold-increase/decrease compared to the control cell values (A: cells not treated with MK-4 or Fsk, B: cells treated with MK-4 for 0 min). Values with different letters are significantly different at *p *< 0.05.

**Figure 8:**
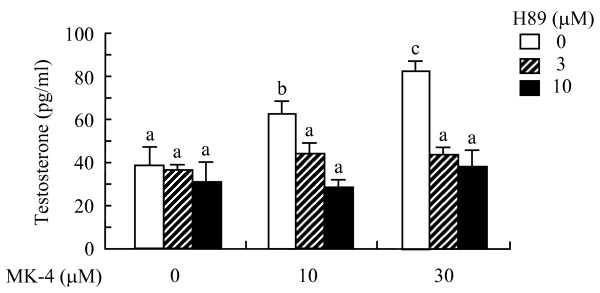
**Treatment with PKA inhibitor abolishes the enhancement of testosterone production by menaquinone-4 in I-10 cells**. I-10 cells were treated with menaquinone-4 (MK-4) and protein kinase A inhibitor H89 simultaneously for 24 h, and then testosterone levels in the culture medium were determined by ELISA. Data are represented as means ± SE (n = 3). Values with different letters are significantly different at *p *< 0.05.

The results are expressed as mean ± standard error (SE). Data in Table 1, Figures 1B, C, and 6B were analyzed using the Student's t test. The data in Figures 2, 3A, C, 4, 5, 6A, 7, and 8 were analyzed statistically by one-way analysis of variance (ANOVA), and multiple comparisons were performed using Tukey's test. The time-course data of plasma testosterone concentrations in samples obtained from the tail vein (Figure 1A) were analyzed by two-way repeated measures ANOVA followed by Tukey's multiple-comparison test. Data in Figure 3B were analyzed by two-way ANOVA, and multiple comparisons were performed using Tukey's test. Statistical analyses were performed using the StatcelQC program (OMS Publishing, Saitama, Japan) and SigmaPlot 12 (Systat Software, Inc., Chicago, IL).

**Table 1:** Vitamin K_1 _and menaquinone-4 concentrations in liver and testis

Organ	Liver		Testis	
	
	**Vitamin K**_**1**_	Menaquinone-4	**Vitamin K**_**1**_	Menaquinone-4
Cont (n = 8)	212.7 ± 20.72^a^	0.634 ± 0.529	7.331 ± 1.621	189.5 ± 8.823
MK-4 sup (n = 7)	77.82 ± 6.415**	2016.8 ± 359.1**	1.481 ± 1.516*	995.9 ± 111.6**

^a^Each value is the mean ± SE (pmol/g tissue). Values are significantly different from those of the Cont group at *p < 0.05, **p < 0.01.

## Results

### Effect of menaquinone-4 supplemented diet on testosterone level in rat testis

Male Wistar rats were fed either a control (Cont) or MK-4-supplemented (MK-4 sup) diet for 5 weeks. After experimental feeding, the overall body weights and relative weights of the liver and testis were not significantly different between the groups (data not shown). Table 1 shows vitamin K concentrations in the liver and testis as determined by fluorescence-HPLC, which illustrates that vitamin K levels in these tissues were strongly affected by the different diets. Specifically, the amount of vitamin K_1 _in tissues from the MK-4 sup group was significantly lower than that in the Cont group, and as expected, MK-4 levels were significantly higher in the MK-4 sup group when compared to that of the Cont group. Testosterone levels in plasma obtained from the tail vein were higher in the MK-4 sup group beginning from the second week of the experiment until the end of the experiment (Figure 1A). Increased plasma testosterone levels in the MK-4 sup group were statistically significant compared with those of the control, and testosterone levels in MK-4 sup group at weeks 3 and 5 were significantly higher than those of Cont group. At the end of the experimental feeding period, testosterone levels in the testis were significantly increased in the MK-4 sup group, although plasma levels of luteinizing hormone (LH) remained unchanged (Figures 1B and 1C). Therefore, an MK-4-supplemented diet can enhance testosterone production in the testis in a manner that is independent of LH.

### Effect of menaquinone-4 on testosterone production in mouse testis tumor cells (I-10 cells)

Mouse I-10 cells have a phenotype similar to that of Leydig cells in that they synthesize and secrete testosterone, which is dependent on intracellular cAMP levels [14]. Testosterone levels in the cell culture medium after addition of dibutyryl cAMP (db-cAMP, 3 μM) were significantly increased compared to control cells that were not treated with db-cAMP (Figure 2A). In addition, treatment of I-10 cells with MK-4 in the presence of db-cAMP was found to significantly enhance testosterone secretion into the culture medium, and the maximum enhancement of secretion was observed when 30 μM MK-4 was present in the medium. Leydig cells secrete testosterone in a diffusive manner [15]; therefore, MK-4 directly enhances testosterone synthesis in the cells rather than only affecting release of testosterone from the cells. Because MK-4 induces apoptosis in several types of tumor cells [16] and osteoclast cells [17] and inhibits growth of hepatocarcinoma cells [18,19], we evaluated the effect of MK-4 on I-10 cell growth and viability. Our results showed that MK-4 did not promote cell growth but rather inhibited I-10 cell growth in a dose-dependent manner (Figure 2B). Therefore, enhanced testosterone secretion stimulated by MK-4 treatment was not due to an increase of the number of cells present.

In addition, MK-4 stimulated testosterone secretion into the culture medium in a dose- and time-dependent manner in the absence of db-cAMP (Figures 3A and 3B), although intracellular levels of testosterone were not changed (data not shown). Previous studies have shown that rat testis-derived R2C tumor cells synthesize and secrete testosterone in a cAMP-independent manner [20]; similarly, we observed that MK-4 stimulates testosterone production in R2C cells without addition of db-cAMP (Figure 3C). Together, these results indicate that MK-4 directly enhances testosterone production in Leydig-like tumor cells independently of intracellular cAMP levels.

### Warfarin does not inhibit enhanced testosterone production by menaquinone-4

Next, we examined whether the formation of Gla proteins is involved in enhanced testosterone production by MK-4. The microsomal enzyme γ-glutamylcarboxylase is responsible for Gla formation and requires the reduced hydroquinone form of vitamin K as a cofactor. Warfarin is an antagonist of vitamin K epoxide reductase, which generates the reduced form from the oxidized or epoxide forms of vitamin K. Thus, warfarin interferes indirectly with Gla formation [21]. When I-10 cells were treated with warfarin in the absence of MK-4, the testosterone levels in the culture medium were modestly increased but not significantly different from control cells (Figure 4). In the presence of MK-4, warfarin did not inhibit the MK-4-stimulated testosterone production, indicating that the stimulation of testosterone production in I-10 cells by MK-4 is not mediated by the formation of Gla proteins.

### Menaquinone-4, but not vitamin K_1_, enhances testosterone production in I-10 cells

We also examined whether vitamin K analogues other than MK-4 stimulate testosterone production and found that vitamin K_1 _does not enhance testosterone production in I-10 cells (Figure 5A). Because vitamin K_1 _may not be incorporated into the cells, we determined the cellular concentration of vitamin K by fluorescence-HPLC after treating the cells with vitamin K_1 _for 3 h; vitamin K_1 _was incorporated into the cells at one-fifth the concentration of MK-4 present (Figure 5B). These results demonstrate that MK-4 is well-incorporated into I-10 cells and may stimulate testosterone production in steroidogenic tumor cells, whereas vitamin K_1 _is incorporated in I-10 cells less than MK-4 and likely has no stimulatory effect on testosterone production in these cells.

### Menaquinone-4 enhances testosterone production through the activation of protein kinase A

CYP11A is the rate-limiting enzyme for testosterone synthesis in Leydig cells. We determined the expression levels of CYP11A and StAR proteins in I-10 cells treated with MK-4. CYP11A levels were significantly higher in cells at 1 h and 3 h post-MK-4 treatment compared to levels in control cells (Figure 6A), and tended to be higher at 9 h post-treatment; no difference of CYP11A levels was found at 24 h post-treatment (data not shown). The StAR expression levels in the cells were unchanged after MK-4 treatment (Figure 6A). Furthermore, CYP11A expression levels in the testis from rats fed a MK-4-supplemented diet for 5 weeks tended to be higher than those of the control rats (Figure 6B). These results indicate that MK-4 may enhance testosterone production via upregulation of CYP11A.

It is well known that expression and activity of CYP11A is regulated by protein kinase A (PKA). Vitamin K stimulates the activities of PKA in various cultured cell types although the detailed mechanism of this activity has not been elucidated [22-24]. Thus, we examined whether MK-4 enhances testosterone production via activation of PKA. Phosphorylation levels of the catalytic subunit of PKA (p-PKA) in I-10 cells were significantly increased by MK-4 treatment at the 2 h and 3 h time points (Figures 7A and 7B), while total PKA levels were not changed (data not shown). In addition, phosphorylation levels of CREB (p-CREB), a typical substrate of PKA, were significantly increased by treatment with MK-4 after 3 h (Figure 7B). Both p-PKA and p-CREB levels were higher in I-10 cells at 9 h post-treatment (data not shown). Conversely, enhanced testosterone production induced by MK-4 was abolished by treatment with H89, a specific inhibitor of PKA (Figure 8). Together, these results indicate that MK-4 increases testosterone production in I-10 cells by upregulating CYP11A expression through the activation of PKA.

## Discussion

Vitamin K plays important roles in the blood coagulation system and in bone formation. However, the tissue distribution of vitamin K is not restricted to only the liver and bone, and is also found in the brain, pancreas, and gonads [11,25]. Specifically, analyses of vitamin K subclasses in rodent brains and testes revealed that MK-4 is the most common form of vitamin K in these tissues. MK-4 is obtained from the diet as well as from the biological conversion of other vitamin K analogues [3-8]. Novel functions of MK-4 have recently been reported [16-18,23,24,26-30], but the physiological significance of vitamin K conversion to MK-4 has not been well characterized. To determine the function(s) of MK-4 in the testis, we previously analyzed the changes of gene expression in the testis of rats fed a vitamin K-deficient diet in order to reduce the levels of MK-4 in the testis [11]. We identified a positive correlation between mRNA expression levels of CYP11A and concentrations of MK-4 in the testis and found that plasma and testis testosterone levels were significantly reduced under vitamin K-deficient conditions without altering the levels of plasma LH secreted from the pituitary gland. These results suggested that MK-4 may be involved in steroidogenesis in the testis. In our current study, we further examined the effect of vitamin K on testosterone production in the rat testis and testis-derived tumor cells. Rats fed a diet supplemented with MK-4 displayed higher plasma and testis testosterone levels when compared to those of control rats. In addition, MK-4 treatment of I-10 cells enhanced testosterone production accompanied by the activation of PKA and elevation of CYP11A protein levels, indicating a novel function of vitamin K that has not been previously reported.

We found that vitamin K_1 _does not enhance testosterone production, while MK-4 significantly stimulates testosterone production and may play an important role in steroidogenesis. Activation of PKA is crucial for steroid production in Leydig cells [10]; therefore, vitamin K_1 _itself likely does not activate PKA in testis-derived cells. Ichikawa *et al*. showed that MK-4, but not vitamin K_1_, enhances mRNA levels of STC2 and GDF15, proteins whose expression is regulated by PKA in human- and mouse-derived osteoblasts [24]. However, Tsang and Kamei showed that both vitamin K_1 _and MK-4 promote nerve growth factor-dependent outgrowth of neuronal cells, which is abolished in the presence of a PKA inhibitor [22]. Discrepancies in results among experiments may be due to differences of the uptake, stability, metabolism, and solubility of each vitamin K cognate used to treat the cells in the culture medium. Suhara *et al*. studied vitamin K uptake in cells by using stable isotope-labeled vitamin K_1 _and MK-4 and found that MK-4 was taken up more rapidly and was better incorporated into HepG2 and MG-63 cells than vitamin K_1 _[31]. Our results indicate that the apparent incorporation of MK-4 was significantly higher than the incorporation of vitamin K_1 _after a 3 h treatment of I-10 cells (Figure 5B). Thus, uptake and stability of each vitamin K analogue may vary among different cell types. Because MK-4 is converted from other vitamin K analogues in organs and cultured cells, administered vitamin K_1 _may be converted to MK-4, which in turn may stimulate PKA activity. However, we did not detect any MK-4 in vitamin K_1_-treated I-10 cells at 3 h and 24 h post-treatment (data not shown); therefore, vitamin K_1 _may be unable to stimulate testosterone production in I-10 cells. Geranylgeraniol, a side chain structure of MK-4, and its derivative chemicals also enhanced testosterone production in I-10 cells (unpublished data). Unsaturated isoprenyl side chain in MK-4, but not in vitamin K_1_, may be important to enhance testosterone production.

The holoenzyme of PKA is an inactivated hetero-tetramer consisting of two regulatory subunits and two catalytic subunits. After the elevation of intracellular cAMP levels by various extracellular stimuli, cAMP binds to the regulatory subunit and induces dissociation of the catalytic subunits from the tetramer complex. Free catalytic subunits of PKA then phosphorylate themselves and substrates of PKA. MK-4 promotes PKA activation [22-24] via a mechanism that has yet to be fully understood. Vitamin D_3 _modulates intracellular cAMP levels in thyroid cultured cells via activation of nuclear receptor VDR [32], and MK-4 acts as a ligand for nuclear receptor PXR (SXR) [26-29], suggesting that MK-4 regulates gene expression, which leads to PKA activation. However, we did not detect any PXR expression in the I-10 cells. Synthetic vitamin K analogues such as vitamin K_3 _(menadione) and vitamin K_5 _stimulate catecholamine-dependent cAMP levels in primary adipocytes [33]. Both forms of synthetic vitamin K could contribute to the stabilization of cAMP in cells, and so MK-4 may also upregulate intracellular cAMP levels in the testis and I-10 cells, although Otsuka *et al*. showed that MK-4 activates PKA without increasing intracellular levels of cAMP [23]. Similar cAMP-independent activation of PKA has been reported [34-37]. The catalytic subunit of PKA interacts with the NFκB-IκB complex, and its kinase activity is inhibited by IκB. Extracellular stimuli for the activation of NFκB (such as lipopolysaccharides and inflammatory cytokines) and vasoactive peptide endothelin-1 induce phosphorylation and degradation of IκB, and the kinase activity of PKA subsequently emerges [35,36]. MK-4 suppresses the activation of IKK, which phosphorylates IκB for its degradation in the NFκB activation pathway [18,30]. Thus, MK-4 may lead to the dissociation of inhibitory peptides (regulatory subunits of PKA or IκB) from the catalytic subunits of PKA. In our preliminary studies, we found that MK-4 cannot activate purified PKA directly *in vitro*; therefore further study is needed to determine the mechanism of MK-4-mediated stimulation of PKA activity.

## Conclusions

We found that MK-4 stimulates testosterone production in rats and testis-derived tumor cells. Decreased testosterone in blood is frequently observed in elderly males and is considered one of the pathogenic factors of age-related diseases such as cancer, osteoporosis, atherosclerosis, and mental disease. MK-4 is already prescribed as a therapeutic and preventive medicine for osteoporosis in Japan and a few Asian countries. In addition, diets containing large amounts of MK-4 and vitamin K_1_, a precursor to MK-4, may contribute to the reduced risk of age-related diseases by promoting increased testosterone production in the testis.

## Abbreviations

db-cAMP: dibutyryl cAMP; FSK: forskolin; Gla: γ-carboxyglutamate; H89: *N*-[2-(*p*-bromocinnamylamino) ethyl]-5-isoquinoline sulphonamide; LH: luteinizing hormone; MK-4: menaquinone-4; PKA: protein kinase A.

## Competing interests

The authors declare that they have no competing interests.

## Authors' contributions

AI participated in the design of the study; carried out the animal experiments, gene expression analysis, and the measurement of testosterone in plasma and testis; performed statistical analysis, and drafted the manuscript. HS conceived of the study and participated in its design and coordination and also edited the manuscript. NT carried out the measurement of testosterone in plasma and testis. YM performed gene expression analysis in I-10 cells. AO measured vitamin K levels in cultured cells. ZHH carried out the measurement of testosterone levels in the culture medium. YO, TS, TG, and MK participated in the design of the study and edited the manuscript. All authors have read and approved the final manuscript.
